# *BrEXLB1*, a *Brassica rapa* Expansin-Like B1 Gene Is Associated with Root Development, Drought Stress Response, and Seed Germination

**DOI:** 10.3390/genes11040404

**Published:** 2020-04-08

**Authors:** Muthusamy Muthusamy, Joo Yeol Kim, Eun Kyung Yoon, Jin A. Kim, Soo In Lee

**Affiliations:** 1 Department of Agricultural Biotechnology, National Institute of Agricultural Sciences (NAS), RDA, Jeonju 54874, Korea; biotech.muthu@gmail.com (M.M.); rlawnduf@korea.kr (J.Y.K.); jakim72@korea.kr (J.A.K.); 2 Department of Chemical and Biomolecular Engineering, National University of Singapore, 4 Engineering Drive 4, Singapore 117585, Singapore; cheyek@nus.edu.sg

**Keywords:** drought tolerance, cell-wall extension, *Brassica rapa*, expansin-like B1, phytohormones, seed germination

## Abstract

Expansins are structural proteins prevalent in cell walls, participate in cell growth and stress responses by interacting with internal and external signals perceived by the genetic networks of plants. Herein, we investigated the *Brassica rapa* expansin-like B1 *(BrEXLB1)* interaction with phytohormones (IAA, ABA, Ethephon, CK, GA3, SA, and JA), genes (*Bra001852, Bra001958*, and *Bra003006*), biotic (Turnip mosaic Virus (TuMV), *Pectobacterium carotovorum,* clubroot disease), and abiotic stress (salt, oxidative, osmotic, and drought) conditions by either cDNA microarray or qRT-PCR assays. In addition, we also unraveled the potential role of *BrEXLB1* in root growth, drought stress response, and seed germination in transgenic Arabidopsis and *B. rapa* lines. The qRT-PCR results displayed that *BrEXLB1* expression was differentially influenced by hormones, and biotic and abiotic stress conditions; upregulated by IAA, ABA, SA, ethylene, drought, salt, osmotic, and oxidative conditions; and downregulated by clubroot disease, *P. carotovorum,* and TuMV infections. Among the tissues, prominent expression was observed in roots indicating the possible role in root growth. The root phenotyping followed by confocal imaging of root tips in Arabidopsis lines showed that *BrEXLB1* overexpression increases the size of the root elongation zone and induce primary root growth. Conversely, it reduced the seed germination rate. Further analyses with transgenic *B. rapa* lines overexpressing *BrEXLB1* sense (OX) and antisense transcripts (OX-AS) confirmed that *BrEXLB1* overexpression is positively associated with drought tolerance and photosynthesis during vegetative growth phases of *B. rapa* plants. Moreover, the altered expression of *BrEXLB1* in transgenic lines differentially influenced the expression of predicted BrEXLB1 interacting genes like Bra001852 and Bra003006. Collectively, this study revealed that *BrEXLB1* is associated with root development, drought tolerance, photosynthesis, and seed germination.

## 1. Introduction

Expansins (EXPs) are cell wall structural proteins which regulate wall expansion during cell growth [1] and stress responses [2] by weakening the hydrogen bond between cell wall polysaccharides. Emerging studies showed that phytohormones interact with the EXPs to coordinate many physiological and cellular processes of plant growth in response to developmental and environmental stimuli [3,4,5]. Phytohormones, including cytokinin, jasmonate, and gibberellin, induce the expression of EXP superfamily [6]. Exogenous application of IAA can increase expansin activity by inducing plasma membrane H^+^-ATPase activity and/or by reducing cell wall pH [7]. It also influences the expression of other cell wall-related genes [8]. EXPs also participate in abscisic acid-mediated cell growth during drought stress [7]. Enhanced expansin activity could contribute to drought-resistant/tolerant by maintaining higher cell turgor and greater cell wall extension during stress conditions [7].

Drought is a combinatorial abiotic stress condition, and the drought responses of plants are regulated by a complex network comprised of several physiological, molecular, and structural factors. The members of expansin superfamily play a role in plant adaptation to various abiotic stresses, including drought stress. Recently, Lenk et al. [9] showed that expression patterns of EXPs and other cell wall-associated genes are crucial in determining drought tolerance level. Genetic approaches altering the expression of expansin multigene families could contribute to stress tolerance against oxidative [10], high salinity [11], heat [12], cold [13], and drought [2,14] stresses. In resurrection plants, drought tolerance is associated with enhanced expansin activity, which has often coincided with a remarkable increase in cell wall extensibility [15]. The cell-wall-enabled protection against stress is possible by elevated EXPs and xyloglucan endotransglucosylase/hydrolase (XTH) that maintains cell wall plasticity [16]. Besides, EXPs can increase the antioxidative properties, photosynthesis rate, and reduce the structural damages to acquire heat stress tolerance in plants [12]. Similarly, root cell elongation and turgor pressure by enhanced expansin activity help plants tolerate drought conditions [17,18]. The high salinity impairs the ability of the cell walls to expand, a process that is under the control of cell wall-located expansin proteins [11]. Therefore, expansin mediated restoration of cell wall expansion processes can be useful to effectively alleviate high salinity stress impacts on cells. Abiotic stresses, including salinity, drought, and cold results in the accumulation of reactive oxygen species, which is the main cause of oxidative stress. However, studies showed that EXPs enhance the activity of cell wall-bound peroxidases to impart stress tolerance by alleviating the oxidative stress damages in cells [10]. Additionally, root architecture is a key feature in determining environmental stress adaptability and crop productivity [19]. Understanding of root development revealed that EXPs involve in primary and lateral root growth during salt and drought environments [20]. Furthermore, EXPs were shown to be involved in micropylar endosperm weakening and radicle growth in seed germination processes [15].

Until now, 53 *EXPs* were annotated in *Brassica rapa* genome [21], and very few of *BrEXPs* were implicated in plant growth and development [22] and stress responses despite its diversity and multiple functionalities. The present study investigates expansin-like B1 gene interaction with a variety of internal and external stimuli and its role in drought tolerance. Our study revealed that the expression of *BrEXLB1* was significantly altered in response to drought, ABA, IAA, ethylene, salt stress, and developmental stages. We further investigated the *BrEXLB1* implications associated with drought stress tolerance in *B. rapa* by overexpressing sense and antisense *BrEXLB1* transcripts. This study revealed that *BrEXLB1* overexpression enhances tolerance level, root growth, and photosynthesis rate under drought stress conditions.

## 2. Materials and Methods

### 2.1. Plant Materials, Growth Conditions, and Treatments

*B. rapa* cv. Chiifu inbreed lines, Turnip mosaic Virus (TuMV)-C4 susceptible (06VR-930, VCS3M-118), and TuMV-C4 resistant (06VR-939, VCS3M-260) DH lines were used for this study. The experimental seedlings (3-week-old) were grown in soil-containing plastic pots, maintained at the growth chamber under continuous light (80 μmol m^−2^ s^−1^) with 16 h/8 h photoperiods, 25 °C temperature, and 70% relative humidity before treatments. For *Pectobacterium carotovorum* infection, 10 μL of *P*. *carotovorum* suspension comprised of approximately 6.15 log_10_ colony-forming units (per milliliter) was inoculated in leaves. Three or four leaves of TuMV resistant, susceptible cultivars were rub inoculated using the TuMV-C4 suspension (along with carborundum powder) and sampled after the first, second, and the third week of infection. For clubroot disease, 1 mL of spore suspension of *Plasmodiophora brassicae* was applied to the stem base of seedlings and sampled at different times (days 10, 27, and 35). The leaf, root samples of treated and the controls were immediately frozen with liquid nitrogen and used for RNA extraction, cDNA synthesis, and subsequent analysis with microarray. The microarray hybridization reaction and data analysis were performed by GGBIO (http://www.ggbio.com) according to the manufacturer’s instructions (NimbleGen Inc., Madison, WI, USA; GenePix scanner 4000B (Axon, Scottsdale, AZ, USA)).

### 2.2. Phytohormones and Abiotic Stress Responses of BrEXLB1 at Different Times

Phytohormones namely indole-3-acetic acid (IAA), abscisic acid (ABA), ethylene (Ethephon), cytokinin (CK), gibberellic acid (GA3), salicylic acid (SA), and jasmonic acid (JA) (each at 100 μM concentration) were added to 8-day-old seedlings growing in hydroponic cultures (*n* = 3). The cultures were maintained at the growth chamber (80 μmol m^−2^ s^−1^ light, 16-h light/8-h dark photoperiod, and 25 ± 0.5 °C temperature) with gentle shaking (60 rpm) for a day. Seedlings grown in MS liquid medium without hormones were used as control. The entire seedlings from all the treatments were harvested, frozen in liquid nitrogen, and stored at −80 °C. Concurrently, for imposing abiotic stresses, NaCl (250 mM; salt), hydrogen peroxide (10 mM; oxidative), D-mannitol (250 mM; osmotic), and polyethylene glycol 6000 (4% *w/v*; drought) were added to the hydroponic cultures and sampled at 0, 0.5, 1, 2, 3, and 6 h after incubation. The hydroponic cultures grown at 4 °C were sampled at different times (0, 0.5, 3, 12, 24, and 48 h) for cold stress. Samples in triplicates for each treatment were used for total RNA extraction, cDNA synthesis, and relative quantification of *BrEXLB1* transcripts using qRT-PCR. The qRT-PCR (CFX96^TM^ Real-Time PCR Detection System (Bio-Rad, Hercules, CA, USA)) was performed with a set of *BrEXLB1* specific primers (FP-TATGGAGAGGGACATGGCAC; RP-CCTCTGGTACTCAACGTCGA) along with AccuPower^®^2X GreenStar Master Mix (Bioneer, Daejeon, Korea). The qPCR condition was as follows: 95 °C for 5 min followed by 40 cycles of 95 °C for 15 s and 56 °C for 30 s. *BrActin2* (FP-CTCAGTCCAAAAGAGGTATTCT; RP-GTAGAATGTGTGATGCCAGATC) was used as an internal control.

### 2.3. Root Phenotyping of Transgenic Arabidopsis Overexpressing BrEXLB1 Sense Transcripts

Wild type and transgenic Arabidopsis seeds overexpressing *BrEXLB1* transcripts (developed previously [21]) were used for this study. The seeds were sterilized and plated on half-strength MS- agar medium along with or without of D-mannitol (at 250, 300, and 350 mM concentrations) in 12 × 12 cm square Petri dishes. After stratification at 4 °C under dark conditions for 3 days, the plates were incubated at a growth chamber (16-h light/8-h dark photoperiod at 23 °C) for 17 days and the root length of all the lines was measured. The laser-scanned microscopic images of root tips (especially elongation zone) of the control and transgenic lines (8-day-old) were obtained using Leica confocal microscope (Leica TCS SP8; Leica Microsystems, Wetzlar, Germany). The growing root tips of 8-day-old seedlings were dipped in propidium iodide (final concentration of 7.5 μg/mL; Sigma-Aldrich, St. Louis, MO, USA) solution for 2–3 min and then washed with distilled water before microscopic imaging.

### 2.4. Development of Transgenic B. rapa Lines Overexpressing Sense and Antisense BrEXLB1 Transcripts

The *BrEXLB1* full-length coding sequence was amplified by polymerase chain reaction (PCR) with gene-specific forward (5′-AATATGAAGACATTTAACGTCTTG-3′) and reverse (5′-GGAATCAAGTAAGTAGAATGTTGG-3′) primers. The PCR amplicons were initially digested with EcoR I restriction enzymes and then, the purified products were inserted (773 bp) into pCAMBIA1390 vector in the transgene orientation between the cauliflower mosaic virus 35S promoter (CaMV35Sp) and the nopaline synthase terminator site (Figure S1). The resultant binary vectors, pCAMBIA1390::35S-Pro+*BrEXLB1* sense (OX) and pCAMBIA1390::35S-Pro+*BrEXLB1* antisense (OX-AS) were genetically transformed with *Agrobacterium tumefaciens* (GV3101) into *B. rapa* cv. Dongbu (DB) to develop transgenic *B. rapa* lines according to the methods of Muthusamy et al. [22]. Cultivar, DB was chosen for its higher transformation efficiency over Chiifu in this study.

### 2.5. Genotyping and Identification of Transgene Integration Sites in Transgenic B. rapa Lines

The genomic DNA was extracted from T1 transgenic lines of OX and OX-AS for genotyping. To amplify insert, PCR was performed with PCR premixture (Solgent, Daejeon, Korea) and primers with the binding site in P35S (P35SR-5′-CGTTCCAACCACGTCTTCAA-3′) and Tnos (Tnos43R-5′-CCGGCAACAGGATTCAATCT-3′) of the transgene expression cassette in a 20-µL reaction mixture. The PCR conditions were as follows: 95 °C for 5 min followed by 20 cycles of 94 °C for 40 s, 55 °C for 30 s and 72 °C for 1 min, and then 20 cycles of 95 °C for 30 s, 57 °C for 30 s, and 72 °C for 1 min, with a final elongation at 72 °C for 5 min. The amplicons were resolved in 1% agarose gel electrophoresis, and the selected amplicons were purified using HiYield Gel/PCR DNA Extraction Kit (RBC Bioscience, New Taipei City, Taiwan) before sequencing by the ABI3730XL DNA Analyzer (Applied Biosystems, Foster City, CA, USA) using the P35SR primer.

Furthermore, T-DNA flanking PCR followed by sequencing was carried out to locate the transgene integration site in the *B. rapa* genome. For this purpose, 500 ng of genomic DNA of selected transgenic plants were digested with Hinc II restriction enzyme, ligated with adapter sequences and then used as a template for PCR reactions as described in Figure S2. The amplicons were sequenced by the ABI3730XL DNA Analyzer, and the sequences were annotated using the NCBI-BLAST tool.

### 2.6. Phenotyping of Transgenic Lines During Irrigated and Drought Stress Conditions

*B. rapa* transgenic (OX, OX-AS) and non-transgenic (DB) seedlings were transferred from in vitro to compost soil (cocopeat (65–70%), peat moss (8–12%), vermiculite (10–14%), zeolite (3–5%), and perlite (5–8%)) under greenhouse conditions. The seedlings were maintained at 25 °C/16-h light during the day, and 22 °C/8-h dark during the night, for three weeks. Drought stress was imposed by withholding the irrigation for 10 consecutive days. A portable SM150T Soil Moisture meter (Delta-T Devices, Cambridge, Great Britain) was used to monitor the soil moisture content before and after drought stress imposition. At the start of the treatment, the soil water content measured to be around 45–50% while it was 1–2% at the end of the stress period. Furthermore, the soil moisture content of the stressed plants was restored to 45–50% for a day and the phenotypic parameters such as plant recovery rate, survivability, and the number of senescent leaves were measured.

### 2.7. Measurement of Chlorophyll a Fluorescence (OJIP Transients)

The 3-week-old transgenic (OX, OX-AS) and the control plants maintained in greenhouse conditions (22/18 °C, 16/8 h light/dark photoperiod) were subjected to 10 days of progressive drought stress. On the day 8, the fully expanded, top leaf from three plants of each line was dark-adapted using clamps for about 30 min. The standard non-imaging OJIP kinetics was measured using the FluorPen FP-110 device (Photon Systems Instruments, Brno, Czech Republic) in the dark-adapted leaves at 455 nm and saturating light pulse of 3000 μmol m^−2^ s^−1^. All the induced fluorescence (OJIP) parameters were recorded and exported to Microsoft Excel (Table S1). The mean values and standard errors derived from three plants of each line were used separately to compare maximum quantum yield of primary chemistry in PS II (Fv/Fm), initial fluorescence (Fo), performance index (Pi-Abs), absorption per reaction center (ABS/RC), and dissipation per reaction center (DI_0_/RC), which are considered as stress tolerance indicators.

### 2.8. Relative Quantification of BrEXLB1 Interacting Genes in Transgenic Lines and BrEXLB1 Phylogenetic Relationship with Other Species

The BrEXLB1 interacting protein partners were predicted (Figure S3) using the protein–protein interaction network prediction tool, string-db at www.string-db.org. All the interacting protein and gene sequences were retrieved from the Brassica database (www.brassicadb.org/brad/searchGene/php/) using gene names. A set of specific primers for few interacting genes were designed at NCBI primer-BLAST, and their expression profiling was done in *BrEXLB1* sense, antisense overexpressors, and nontransgenic *B. rapa* plants using qRT-PCR. Five μg of total RNA (free of genomic DNA content) extracted from transgenic and the control lines (RNeasy Plant Mini Kit (Qiagen, Hilden, Germany)) was reverse-transcribed in a 20 μL reaction mixture using amfiRivert cDNA Synthesis Platinum Master mix (GenDepot, Katy, TX, USA) according to the manufacture’s protocols. Quantitative PCR reaction for each gene was set up with 20 μL reaction mixture containing 60 ng of total cDNA, specific primers (specific to Bra001852, Bra001958, and Bra003006), and AccuPower^®^2X GreenStar Master Mix (Bioneer) in a CFX96^TM^ Real-Time PCR Detection System (Bio-Rad). *BrAct2* (forward: 5‘-TGGCATCACACTTTTCTACAA-3‘; reverse: 5‘-CAACGGAATCTCTCAGCTCC-3‘) was used as an internal control, and the expression of each gene was normalized with the control lines for measuring relative expression. The amino acid sequences of BrEXLB1 (248 AA in length) and EXLB1 of 57 other species were retrieved from the UniProt database (http://www.uniprot.org). The phylogenetic relationship was assessed using the maximum likelihood tree method (1000 bootstrap replicates) in MEGA ver.7 tool. The primer sequences and its annealing temperatures were listed in Figure S3.

### 2.9. Statistical Analyses

All the treatments mentioned in this study had at least three independent biological and technical replicates. By default, the mean value of three replicates was considered for data analysis, and the standard error was presented as error bars in graphs drawn using GraphPad Prism 5 or Microsoft excel tool. The GraphPad Prism 5 tool was used to perform one-way analysis of variance (one-way ANOVA) followed by Tukey′s HSD test to assess the significant variation that exists between the treatment and the control groups. Statistics by ANOVA test are shown; * *P* < 0.05, ** *P* < 0.001, *** *P* < 0.0001, and NS, no significance.

## 3. Results

### 3.1. Differential Expression of BrEXLB1 to Hormone and Stress Treatments

**Figure 1:**
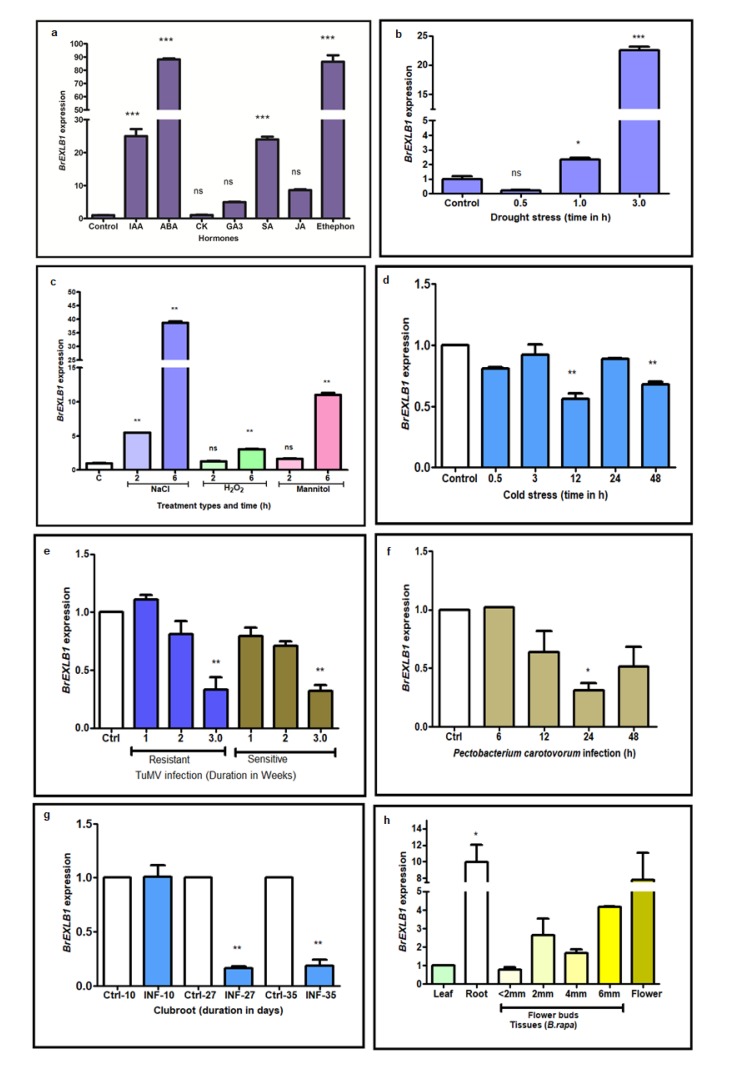
Differential expression pattern of *BrEXLB1* in response to different hormones, abiotic, and biotic stress conditions. The graphs depict the relative quantification of transcripts of *BrEXLB1* during the exogenous application of hormones (**a**) such as indole-3-acetic acid (IAA), abscisic acid (ABA), cytokinin (CK), gibberellin (GA3), salicylic acid (SA), jasmonic acid (JA), ethylene (Ethophen), drought stress (**b**); salt stress (NaCl), oxidative stress (H_2_O_2_), and osmotic stress (D-Mannitol) (**c**); cold stress (**d**); Turnip mosaic Virus infection (TuMV) (**e**); *Pectobacterium carotovorum* infection (**f**); clubroot disease (**g**); and tissue-specific expression in *Brassica rapa* (**h**) plants (** represents statistical significance). INF denotes infection; C or Control or Ctrl denotes wild type plants.

The exogenous application of IAA, ABA, SA, and ethylene on *B. rapa* induced the expression of *BrEXLB1* significantly over the control plants (Figure 1a). The similar exogenous application of phytohormones such as CK, GA3, and JA did not influence the expression of *BrEXLB1* significantly. Further *BrEXLB1* responses to abiotic (drought and salt stress), biotic (TuMV infection, *P.carotovorum*, and clubroot disease), and other secondary stresses (osmotic and oxidative) were investigated in this study. The results revealed that drought stress, high salinity, osmotic, and oxidative stress conditions upregulated the *BrEXLB1* expression significantly at transcriptional level (Figure 1b,c). Among biotic stress conditions, TuMV infection (3 h post-inoculation in TuMV resistant and sensitive cultivars), *P. carotovorum* (24 h post-inoculation), and clubroot disease (27 days and above) caused a significant reduction in *BrEXLB1* expression (Figure 1e–g). Interestingly, cold stress (4 °C for 48 h) reduced the expression level of *BrEXLB1* significantly compared to the controls (Figure 1d). Among the tissues, prominent expression was observed in roots indicating the possible role in root architecture/root growth. During progressive biotic stresses, the BrEXLB1 activity was reduced, suggesting inverse relationships between these two attributes. As indicated in the hormonal response, the expression of *BrEXLB1* was more pronounced in abiotic stresses (except for the cold stress) than biotic stress conditions. The drought, salinity, oxidative, and osmotic stresses significantly upregulated the *BrEXLB1* expression. Interestingly, cold and biotic stress conditions either downregulated the expression of *BrEXLB1* during stress conditions or showed no difference with that of controls.

### 3.2. BrEXLB1 Associated with Root Growth and the Size of the Elongation Zone in Growing Roots

**Figure 2:**
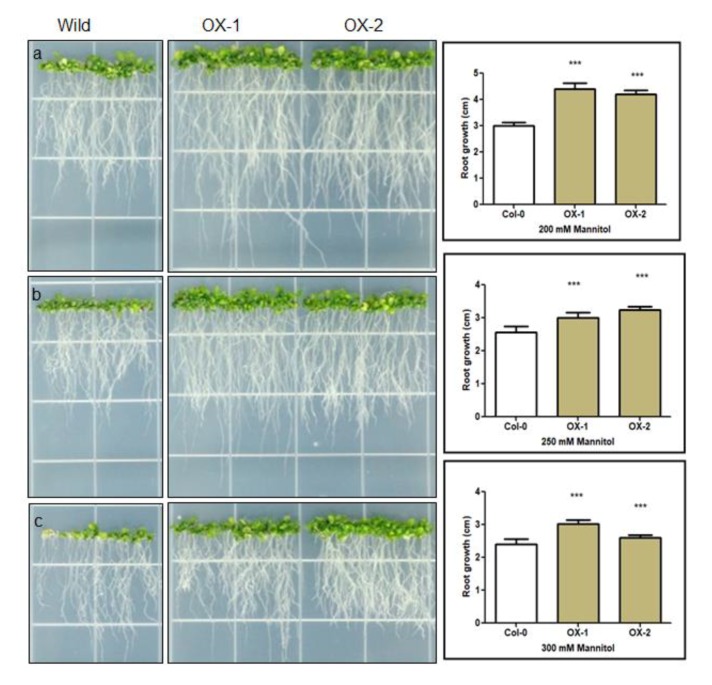
Comparison of root phenotypes of transgenic Arabidopsis lines overexpressing *BrEXLB1* (OX-1 and OX-2) and wild (Col-0) in the presence of 200 mM (**a**), 250 mM (**b**), and 300 mM of D-Mannitol (**c**) in Murashige and Skoog (MS) agar plates for 17 consecutive days (*** represents statistical significance).

Our previous study has shown that *BrEXLB1* is preferentially expressed in roots, carpels, and seeds [21]. Literature showed that root architecture/development is associated with the abiotic stress response of plants. Under mannitol induced drought/osmotic stress conditions, the root phenotypes of wild and OX lines have differed significantly (Figure 2). In the presence of 200 mM mannitol in the growth medium, the selected lines of OX-1 and OX-2 had better root growth of 4.39 ± 0.16 (SE) and 4.21 ± 0.15 cm, respectively, over the control (3.0 ± 0.14 cm) plants. Although the increasing concentration of D-mannitol (250 and 300 mM) produced negative impacts on the root growth of all the lines, the root length of both transgenic lines appeared to be significantly higher than the wild type lines. The OX-1 lines had the root length of 3.74 ± 0.16 cm in presence of 250 mM mannitol, while the same lines had the root length of 3.02 ± 0.13 cm under 300 mM mannitol conditions. Similarly, OX-2 lines had 3.74 ± 0.16 cm and 3.02 ± 0.13 cm respectively in the presence of 250 and 300 mM mannitol over the wild type lines which had 2.55 ± 0.18 and 2.41 ± 0.15 cm root length under similar conditions.

**Figure 3:**
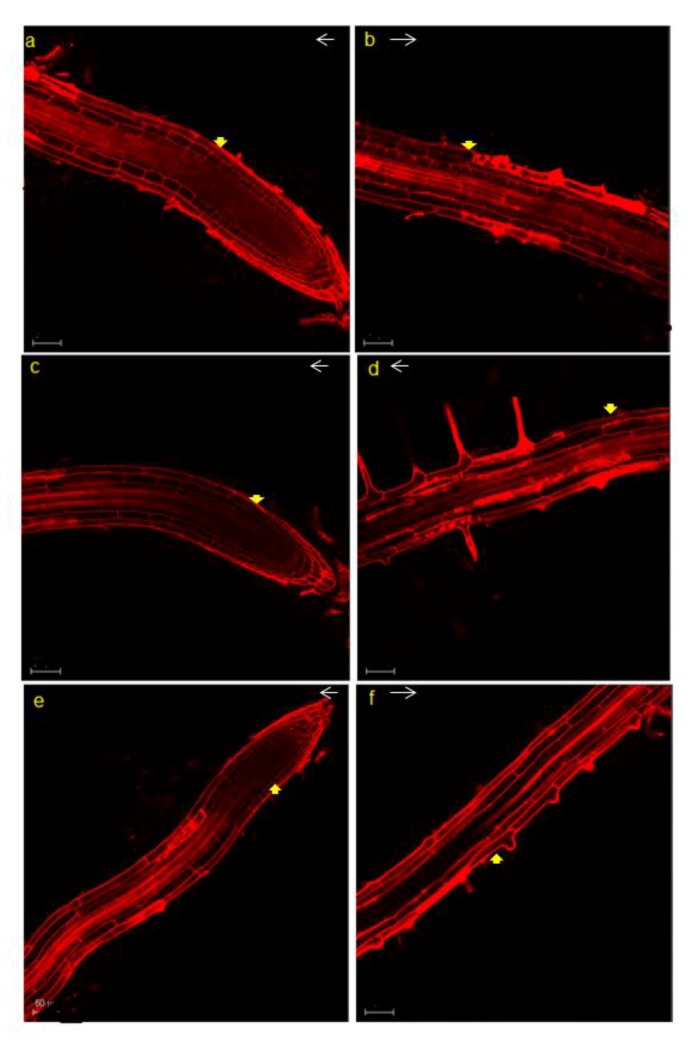
Comparison of root epidermal and internal cell layers in samples collected from *BrEXLB1* overexpressing transgenic Arabidopsis (OX) and wild type lines. (**a**), (**c**), and (**e**) represent root tips of wild, OX-2, and OX-1, respectively; while (**b**), (**d**), and (**f**) represent section followed by root tips. White arrows denote the direction of the root tip. The yellow arrows in (**a**,**c**,**e**) and (**b**,**d**,**f**) respectively, denote the start and end of the elongation zone in the roots. Scale bar = 200 μm.

To further understand the role of *BrEXLB1* mediated cell expansion activity, root architecture (e.g., the elongation zone) was analyzed using confocal microscopy. The microscopic images revealed that the size of the elongation zone (with or without transition zone) in transgenic lines (OX) appeared to be larger than wild type lines (Figure 3). The increased zone of cell elongation supposed to negatively affect the length of meristematic region of roots, which is evident from the transgenic lines. Contrastingly the length of meristematic zones in wild lines was relatively longer than transgenic lines. The results showed that overexpression of sense *BrEXLB1* positively associated with increased length of root cell elongation zone and primary root growth.

### 3.3. BrEXLB1 Overexpression Inversely Associated with Seed Germination Efficiency

Our previous study showed that *BrEXLB1* associated with seed development [21]. In this study, we investigated the influence of *BrEXLB1* overexpression in transgenic Arabidopsis seed germination. The germination efficiency of transgenic seeds was tested with or without gibberellins (GA) over a period of 5 days. The comparative analysis showed that the transgenic seeds (OX-1 and OX-2) have a germination percentage of 56 and 68% under normal conditions, which is relatively lesser than Col-0 (88%). Under GA treatment, the germination efficiency of transgenic seeds (59–78%) and non-transgenic controls (97%) was increased, however, a significant difference in germination efficiency was observed between the transgenic and control seeds. Similarly, germination of *B. rapa* OX lines was completely inhibited in the presence of 20% of polyethylene glycol (PEG 6000) solution, while seeds of OX-AS lines produced 100% seed germination rate. These results indicate that overexpression of *BrEXLB1* is inversely associated with seed germination under both normal and drought conditions.

### 3.4. Identification of T-DNA Integration Sites and Drought Phenotyping of BrEXLB1 Sense and Antisense Overexpressing B. rapa Lines

**Figure 4:**
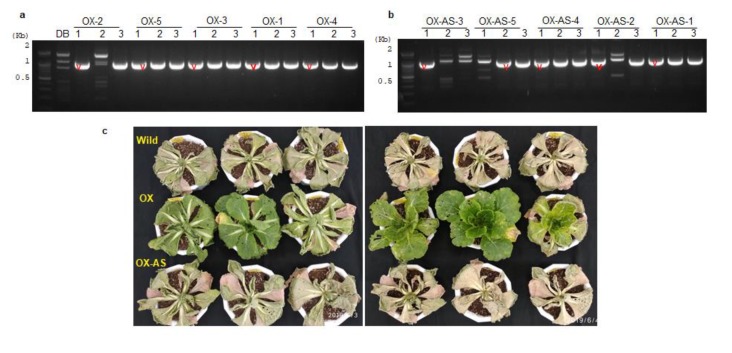
Transgene expression in transgenic *B. rapa* lines and their drought phenotypes. (**a**,**b**) represent the transgenic lines overexpressing BrEXLB1 sense (OX) and antisense transcripts (OX-AS) in *B. rapa* lines, while (**c**) represents the stay-green phenotypes of transgenic lines subjected to 10 days of consecutive drought stress followed by recovery. The red color marks in (**a**,**b**) indicate the transgenic lines, chosen for *BrEXLB1* sequence confirmation.

The transgenic lines were genotyped using PCR (Figure 4), and sequencing as described in the methods section. Additionally, the T-DNA integration sites were identified from T-DNA flanking region sequencing from the selected transgenic plants. In OX-AS transgenic lines, T-DNA found to be integrated at intron (Chr A05: 21808504...21808763) of Bra034868 (designated as OX-AS-2 lines), which is encoding for outer membrane OMP85 family protein. Similarly, in OX lines, T-DNA containing transgene found to be integrated at multiple loci, including 0.18kb 5’ upstream of Bra035051 (Chr A07: 21964723..21964955), intron of Bra015086 (Chr A07: 3590653..3590364), intergenic region of Bra034883 (Chr A08: 5730071..5730299), 5′ upstream of unknown protein (Chr A01: 23795482..23795610), and 0.31kb upstream of unknown protein (Chr A1: 23795482..23795610). These transgenic lines were named as OX-1, OX-2, OX-3, OX-4, and OX-5, respectively. The qRT-PCR based expression profiling of *BrEXLB1* transcripts in these lines revealed that OX-1—followed by OX-2, OX-3, OX-4, and OX-5, respectively—had a high expression of 100.49, 23.37, 10.59, 9.09, and 6.4 (in folds) in comparison with the control lines. Similarly, OX-AS-1, OX-AS-2, OX-AS-3, OX-AS-4, and OX-AS-5, respectively, had the overexpression of 257.4, 100.6, 86.6, 21.6, and 3.8 folds over the control lines. Transgenic lines with the highest expression of *BrEXLB1* transcripts (OX-1 and OX-AS-1) were selected for further studies.

The progressive drought stress was imposed by suspending the irrigation for 10 consecutive days. The drought stress differentially influenced the phenotypes and recovery rate of transgenic and nontransgenic plants. Consistent with *BrEXLB1* sense and antisense expression, OX lines recovered well with approximately 20% leaf senescence, while lines of *BrEXLB1* antisense overexpressors and controls completely wilted under stress and had 100% leaf senescence rate. The drought recovered OX lines, after a week, were incubated at 4 °C for 40 days (vernalization) to initiate flowering and then maintained at the greenhouse for seed production. Of these, seed production in the OX-1 lines with the highest expression of *BrEXLB1* was severely hampered and produced an average of 10 seeds only per plant against 167 seeds in the OX-2 line.

**Figure 5:**
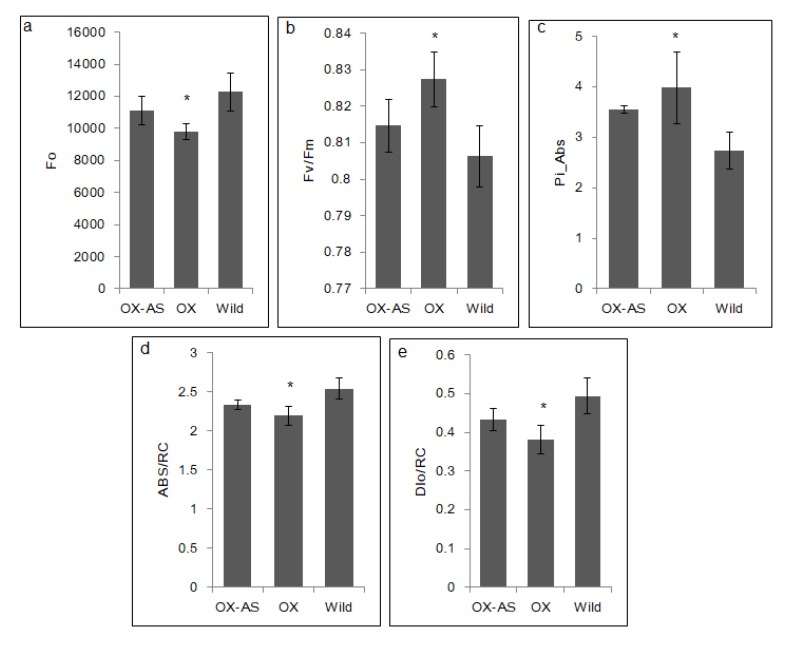
Comparison of chlorophyll fluorescence transients of dark-adapted leaves of *BrEXLB1* sense (OX), antisense (OX-AS), and wild type *B. rapa* plants subjected to 8 days of progressive drought stress conditions. (**a**–**e**) represent initial fluorescence (Fo), maximum quantum yield of primary chemistry (Fv/Fm), performance index (Pi-Abs), absorption per reaction center (ABS/RC), and dissipation per reaction center (DI_0_/RC) in PS II, respectively. * represents the statistical significance.

To evaluate the drought stress impacts on photosystem II (PS II) of OX, OX-AS, and control *B. rapa* lines, the chlorophyll fluorescence induction kinetics (OJIP transients) were measured during drought stress (Figure 5). The OJIP parameters such as maximum quantum yield of primary chemistry in PS II (Fv/Fm), initial fluorescence (Fo), performance index (Pi-Abs), absorption (ABS/RC), and dissipation (DI_0_/RC) per reaction center of OX lines were significantly different from the controls. Fv/Fm and Pi-Abs were relatively higher in OX than that of control and OX-AS. Moreover, Fo, ABS/RC, and DI_0_/RC were relatively lower in OX than that of OX-AS and the controls (Figure 5). Interestingly, differences between OX-AS and the control, and between OX-AS and OX lines were not significant. This chlorophyll a fluorescence kinetics indicated that performance and the state of PS II of OX lines were less affected by drought stress.

### 3.5. Impact of Altered Expression of BrEXLB1 on Downstream Genes

**Figure 6:**
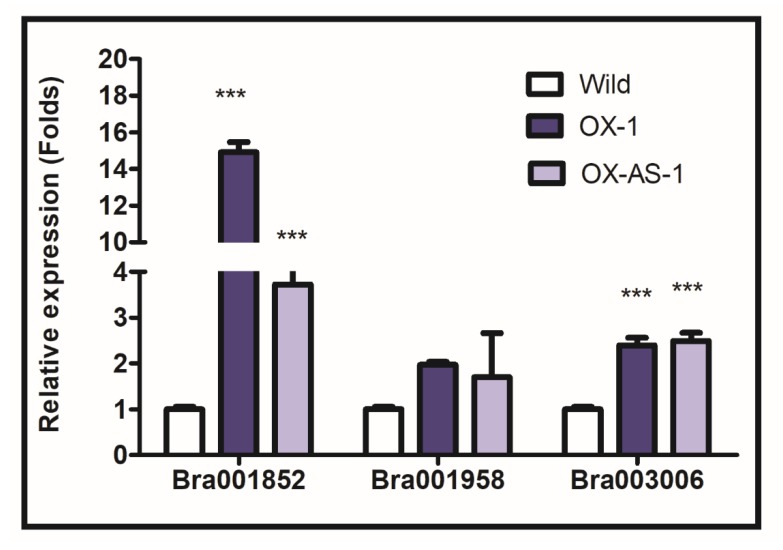
Expression changes of the BrEXLB1 interacting genes in OX-1 and OX-AS-1 transgenic and wild type *B. rapa* plants. *** represents the statistical significance.

To evaluate the impact of altered *BrEXLB1* expression on the expression pattern of BrEXLB1 interacting genes, expression profiling of cell wall-plasma membrane linker protein-encoding gene (Bra001852), non-symbiotic hemoglobin 1 (Bra001958), and a wound response AT5G54170 homologous gene (Bra003006) was done in OX and OX-AS lines by qRT-PCR assay (Figure 6). The expression profiling of these genes showed that their expression was upregulated in both OX and OX-AS lines, but the magnitudes of their expression levels were different. In comparison with the controls, the strongest expression was observed for Bra001852 in OX-1, followed by its expression in OX-AS-1. Interestingly, the expression level of Bra003006 in both OX and OX-AS lines was similar and overexpressed than the controls. However, Bra001958 expression was not statistically influenced by either *BrEXLB1* overexpression in OX lines or *BrEXLB1* suppression in OX-AS-lines. This study showed that the alteration of *BrEXLB1* expression is likely to influence the expression of some of the BrEXLB1 interacting genes.

### 3.6. EXLB1 Phylogenetic Relationships

**Figure 7:**
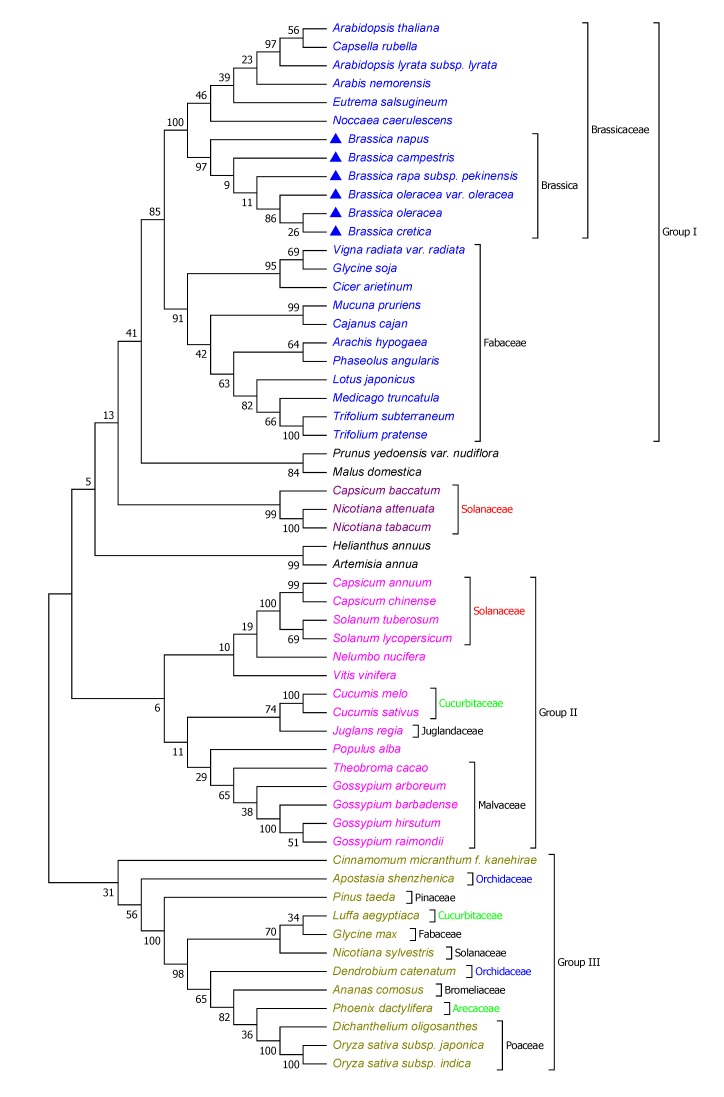
Phylogenetic relationship of EXLB1 of *B. rapa* and other species. The phylogenetic tree was constructed with maximum likelihood algorithm with 1000 bootstrap replicates using MEGA 7.0 tool.

The amino acid sequences of EXLB1 were retrieved from the UniProt database (http://www.uniprot.org). The phylogenetic relationships between EXLB1 amino acid sequences (248 AA in length) of *B.rapa* and other plant species were assessed using the maximum likelihood tree method (1000 bootstrap) in MEGA ver.7 tool (Figure 7). The phylogenetic analysis of 57 species can be classified into three major groups. Group I comprised most of the Brassicaceae members, including Brassicas, *Arabidopsis thaliana,* and Fabaceae (e.g., *Glycine soja*, *Vigna radiata* var. *radiata*). Group II has members, mostly from Solanaceae, Malvaceae, and Cucurbitaceae. Similarly, Group III has 11 families, including Poaceae and Orchidaceae. The phylogenetic relationship of EXLB1 gene of Brassicaceae and Fabaceae indicates that both these family members might have evolved from common ancestors. In Brassicaceae, all brassica members formed a separate clade from other members such as *A. thaliana* and *Capsella rubella*, indicating the Brassica-specific divergence. This result also indicates that BrEXLB1 relatively conserved among Brassica species. As an outgroup Rosaceae (e.g., *Prunus yedoensis* var. *nudiflora* and *Malus domestica*) might distantly be related to Group I members. Group II is mostly represented by Solanaceae and Malvaceae members, and both formed a separate subclade within the group. The EXLB1 relationships in Group III are interesting as it draws members of multiple families and the prediction is fairly significant as indicated by the bootstrap values. It has species from Poaceae, Orchidaceae, Pinaceae, Cucurbitaceae, Fabaceae (*Glycine max*), Bromeliaceae, and Arecaceae families. This study showed some interesting fact that EXLB1 of *Pinus taeda*, *G. max* (L.), *Nicotiana sylvestris*, *Dendrobium catenatum*, and *Oryza sativa* were phylogenetically linked as indicated in group III. Among families, EXLB1 of Solanaceae members seems to have highly diverged during evolution than other families analyzed in this study. It is likely possible that EXLB1 of Brassicaceae underwent unique selection pressures, which was evidenced, as shown in the group I (Figure 7).

## 4. Discussion

### 4.1. Hormonal Responses of BrEXLB1 Indicate Their Participation in Stress Responses

The exogenous application of IAA, ABA, SA, and ethylene significantly upregulated the expression of *BrEXLB1*, suggesting their participation in growth and development and stress responses. However, *BrEXLB1* may not participate in JA, GA3, and cytokinin mediated signaling pathways and stress responses as indicated by its unchanged responses to these hormones. ABA can induce expansin activity mainly by cell wall basification via decreasing plasma membrane H^+^-ATPase activity. On the other hand, IAA application induces expansin activity, mainly due to the decrease of cell wall pH by increasing plasma membrane H^+^-ATPase activity [7]. The IAA treatment with the model plant Arabidopsis significantly induced the expression of three expansins (*EXPA4, EXPA11*, and *EXLA3*) of cell wall-related proteins [1]. Previously, Chen et al. [2] reported that expansin involved in cell wall changes induced by phytohormones, ABA, auxin, ethylene, drought, and salt stresses. In this study, the exogenous application of IAA possibly activates *BrEXLB1* through wall acidification, which degrades the polysaccharide network or induces the cell wall property changes accompanied by cell growth. Similarly, BrEXLB1 interaction with ABA associates them with seed development, germination, stomatal closure, stress-responsive gene expression, shoot growth, circadian clock, abiotic stress responses, including drought and osmotic stresses, and many other cellular and physiological processes [5]. However, the exogenous SA mediated regulation of *BrEXLB1* is in disagreement with the findings of Coqueiro et al. [3], who reported that SA application reduces the expression of expansin and other cell wall-associated genes in sweet orange. Similarly, Nafisi et al. [6] reviewed that phytohormones, including CK, JA, and GA3 induce EXP expression. The role of SA, JA, and ethylene was implicated in cell wall mediated defense signaling network [4,6]. Collectively, our results show that *BrEXLB1* may participate in IAA, ABA, SA, and ethylene mediated signaling networks and other multiple biological processes. EXPs are one of the structural proteins of the cell wall; they regulate wall expansion during cell growth in various tissues and organs [1]. The expansin mediated cell wall extension during abiotic stress conditions like drought is helpful for stress relaxation in plants [23], while its enhanced expression is considered to favor the pathogen evasion/ pathogen attack and cause disease susceptibility [24]. Expansin activity could contribute to drought-tolerance by maintaining greater cell wall extension [7]. Interestingly, in this study, abiotic stress conditions (drought, salt, oxidative, and osmotic) upregulated the *BrEXLB1*, while TuMV, *P.carotovorum* infections, and clubroot disease reduced the expression of *BrEXLB1* indicating their possible positive correlation with abiotic and biotic stress tolerance.

### 4.2. Overexpression of BrEXLB1 is Positively Associated with the Size of the Elongation Zone and Root Growth in Arabidopsis

Cell expansion is an essential component of many plant morphogenetic processes such as cell enlargement, stress response, fruit softening, pollen tube development, root hair growth, and abscission [25]. Drought condition restricts cell extension and cell division, which ultimately reduce plant growth and resulting in economic losses [2]. Plants maintaining root growth under water deficit conditions have a high degree of survival rate. Hence, we analyzed the primary root phenotypes of OX and wild type plants under mannitol induced stress conditions. The results revealed that transgenic lines had better root growth over the control plants and it is consistent with *BrEXLB1* expression. This result indicates that BrEXLB1 would directly or indirectly confer stress tolerance. Furthermore, previous reports revealed that a decrease in cell elongation can lead to drought-sensitive phenotypes [26]. In other words, plants with relatively higher expansin activities under low water potential can be considered as stress-tolerant cultivars/varieties. Therefore, we also analyzed the elongation zones in the root phenotypes of wild and transgenic plants using confocal microscopy. The microscopic dissection of the growing root tip of transgenic and wild lines showed differences in the size of elongation and meristematic zones. The size of the elongation zone is larger in one or more OX lines than wild type plants, while the length of the meristematic zone was relatively smaller than wild type lines. *BrEXLB1* overexpression is consistent with the increase in the size of the elongation zone, thus confirming its role in cell elongation and/or root growth. Previously, Kong et al. [27] suggested that soybean *EXLB1* may be associated with lateral root emergence. Therefore, we also investigated the *BrEXLB1* association with lateral root development by expression profiling of known lateral root marker genes such as *GATA23* and *MAKR4* genes in OX and OX-AS lines (Figure S4) and also comparing the lateral root phenotypes. The results showed that *BrEXLB1* overexpression in OX lines does not affect *GATA23* and *MAKR4* gene expression, which is consistent with their lateral root phenotypes. However, overexpression of *BrEXLB1* antisense transcripts in OX-AS lines significantly reduced the *GATA23* and *MAKR4* expression, although the phenotypes of OX-AS lines look similar to that of control lines. This result show that *BrEXLB1* may not directly participate in lateral root development of 4-week-old *B. rapa* plants or possibly other unknown factors modulate *BrEXLB1* mediated later root development.

### 4.3. Altered Expression of BrEXLB1 Influences the Drought Stress Response, Performance of Photosystem II, and the Expression of BrEXLB1 Interacting Genes

The comparative analysis of *B. rapa* lines under 10 days of progressive drought stress conditions revealed that *BrEXLB1* expression in transgenic lines differentially influences the drought stress responses of plants. The transgenic lines with *BrEXLB1* overexpression produced stay-green phenotypes (100% survival rate) with the least leaf senescence rate of 20% during stress. Conversely, both OX-AS lines and wild types permanently wilted and appeared to have 100% leaf senescence for drought stress. Furthermore, chlorophyll a fluorescence is considered to be a good indicator of stress impacts, especially the PSII (leaf photosynthetic apparatus) system in leaves [28]. Drought stress is known to negatively affect the functions and activity of PSII [29]. Hence, we measured the chlorophyll a fluorescence kinetics (OJIP transients), to determine the level of susceptibility to stress and stress tolerance in the dark-adapted samples of OX, OX-AS, and the controls [30]. The values of Fv/Fm and performance index (Pi-Abs) in OX lines indicated that the function and activity of PS II were fairly maintained during stress conditions than the control and OX-AS lines. Among the tested lines, the Fo, ABS/RC, and DI_0_/RC per reaction center in OX lines was the lowest one suggesting the number of active reaction centers is more than the control and OX-AS lines. The OX lines with these OJIP parameters can be considered as relatively drought-tolerant lines as described previously in rubber tree clones [29]. On the contrary, OX-AS lines neither produced opposite phenotypes to OX nor have statistically significant differences with the control lines. This can be reasoned from the fact that (i) drought induced *BrEXLB1* sense transcripts is not sufficiently countered in OX-AS or (ii) the loss of *BrEXBL1* function in OX-AS is possibly complemented by other expansin members found in *B. rapa* genome, although no concrete evidence is available. Nonetheless, it is confirmed that BrEXLB1 activity is directly or indirectly associated with the maintenance of PS II under drought conditions. Moreover, our study shows that *BrEXLB1* overexpression may negatively affect the seed germination efficiency in Arabidopsis and *B. rapa* lines.

The analysis of expression changes of BrEXLB1 interacting genes in OX and OX-AS lines revealed that altered expression of *BrEXLB1* is associated with expression changes/activity of some of BrEXLB1 interacting genes. In Brassica, their function is mostly unknown. A study dealing with clubroot disease found that expression of Bra001958 and Bra003006 is being upregulated in the resistant genotype of *B. napus* against *P. brassicae* infection, while Bra001852 was found to be suppressed [31]. Hence, we presume that the induced expression of Bra001958 and Bra003006 may favor disease resistance in OX lines. Bra003006 encodes for a lipid-binding domain-START containing protein, while Bra001958 and Bra001852 encode non-symbiotic hemoglobin1 and cell wall-plasma membrane linker protein, respectively. Nonetheless, the studies on Bra001958 homologs found in other plants—including Arabidopsis—participate in cadmium tolerance (cd), detoxification of nitric oxide (NO), and reactive oxygen species, tolerance to abiotic stresses (e.g., salinity and osmotic stress) and pathogen attack [32]. A further in-depth study is essential to understand the role of Bra001852 and Bra003006 in *BrEXLB1* mediated stress responses and growth regulation. However, it is confirmed that *BrEXLB1* is directly or indirectly influences the expression of BrEXLB1 interacting genes.

In earlier studies, *B. rapa*-specific evolutionary dynamics and structural relationships at gene, domain and promoter motif levels were detailed [21,33]. However, the broader phylogenetic relationships of EXLB1 in plant kingdom were scarce. In this study, the evolutionary relationships of 57 plant species comprised of several families were investigated and found that Brassicaceae members evolutionarily related to Fabaceae despite their unique selection pressure followed by divergence. In conclusion, our study demonstrated with *BrEXLB1* overexpression is contributing to enhanced root growth, drought tolerance, and photosynthesis in *B. rapa* by responding to hormones and stress signals.

## Supplementary Materials

The following are available online at https://www.mdpi.com/2073-4425/11/4/404/s1, Figure S1: The simplified version of expression cassette used for Agrobacterium-mediated genetic transformation and transgene (*BrEXLB1*) expression pattern in transgenic *Brassica rapa* lines; Figure S2: The methodology followed in T-DNA flanking sequencing and the list of primers used in this study for identification of transgene integration site; Figure S3: The BrEXLB1 protein–protein interacting prediction network obtained from String-db tools and the list of primers designed for expression profiling of BrEXLB1 interacting genes; Figure S4: qRT-PCR based relative quantification of lateral root marker genes such as *GATA23* and *MAKR4*; Table S1: List of OJIP transient parameters collected from OX, OX-AS lines, and wild type *B. rapa* lines subjected to 8 days of progressive drought stress.
