# Supplementary material for: BrEXLB1, a Brassica rapa Expansin-Like B1 Gene Is Associated with Root Development, Drought Stress Response, and Seed Germination
Source: Genes (Basel). 2020 Apr 8;11(4):404. doi: 10.3390/genes11040404 (PMC7230339; doi:10.3390/genes11040404)
Supplement: Supplementary file 1 [file genes-11-00404-s001.pdf]

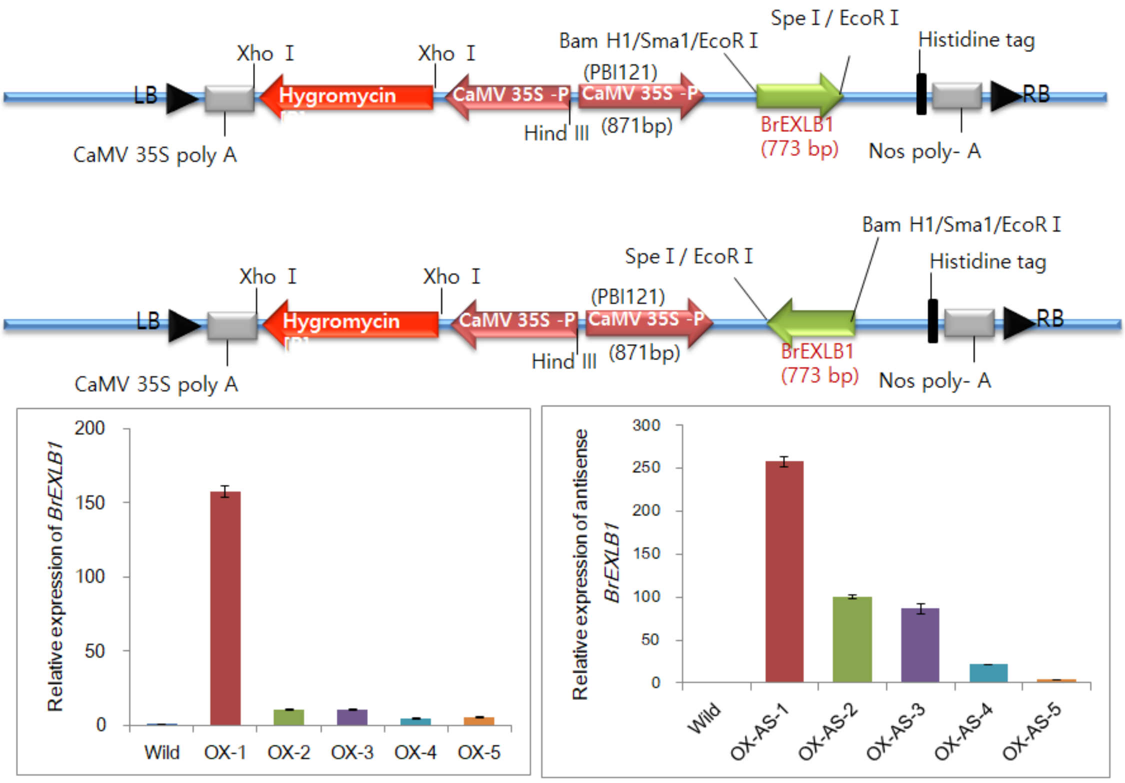


**Figure S1.** The simplified version of expression cassette used for Agrobacterium mediated genetic transformation and transgene (BrEXLB1) expression pattern in transgenic *Brassica rapa* lines.

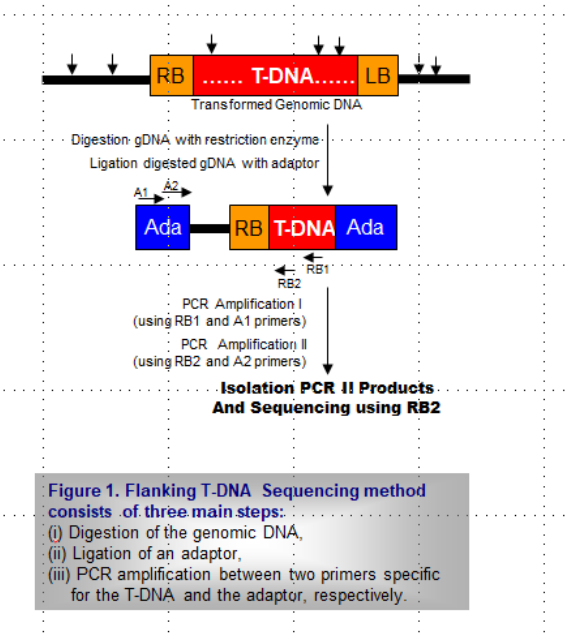


| | 1$^{ST}$ primer | 2$^{nd}$ primer |
|---|---|---|
| LB | HPTII-970F-an70 | HPTII-99i F |
| RB | 2Cam3301_RB1an70 | CamNos_R2 |

**Figure S2.** The methodology followed in T-DNA flanking sequencing and the list of primers used during this study for identification of transgene integration site.

Flanking T-DNA Sequencing method consists of two main steps: first, restriction digestion of the genomic DNA and ligation of an adaptor, and second PCR amplification between two primers specific for the T-DNA and the adaptor, respectively. 500 ng genomic DNA were digested and ligated with 2U restriction enzyme and 5U T4 DNA ligase (Takara, Shiga, Japan) in a 20ul reaction volume. The reaction mixture contained T4 DNA ligase buffer and 50 pmol of the adaptors was incubated at 37 ℃ for 1hr. The first PCR was conducted in a 20 uL reaction containing PCR pre-mixture, 0.5 pmol each primer (Ada1 and LB1 or RB1), and 1 uL digestion/ligation product. PCR were performed in a PTC-200 thermal cycler (MJ Research, Waltham, MA, USA) and conducted as the follows(Ada2 and LB2 or RB2): first denaturation step at 95 ℃ for 5 min, 20 cycles of 30 s at 94 ℃ and 1 min at 72 ℃, followed by a final elongation step at 72 ℃ for 10 min. Second PCR were conducted with 5ul of first PCR product under the conditions of first denaturation step at 94 ℃ for 5 min, 40 cycles of 30 s at 94 ℃, 30 s at 60 ℃, and 1 min at 72 ℃, followed by a final elongation step at 72 ℃ for 10 min. Amplified products were loaded on a 1% agarose gel and the PCR products were purified by HiYield™ Gel/PCR DNA Extraction Kit (RBC Bioscience, Taipei, Taiwan) and sequenced by ABI3730XL using LB2 or RB2 primer.

The list of primers used for T-DNA Flanking sequencing in this study

| S.No | Primer Name | Sequences |
|---|---|---|
| 1. | CAMBIA-RB-an70(2Cam3301-RBan70) | tgaattacaggtgaccagctcgaatttcc |
| 2. | Tnos149F (CamNos-R2) | TGATTAGAGTCCCGCAATTA |
| 3. | HPT_991F | CTCGCCGATAGTGGAAACC |
| 4. | HPT_970F_an70 | CCGTCTGGACCGATGGCTGTGTAGAAG |

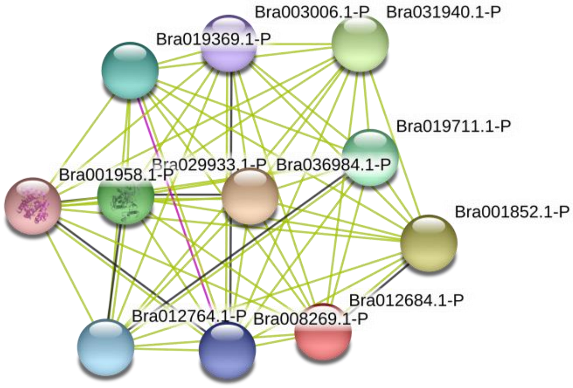


**Figure S3**. The BrEXLB1 protein-protein interacting prediction network obtained from String-db tools and the list of primers designed for expression profiling of BrEXLB1 interacting genes

<u>Details of interacting proteins and their corresponding gene names from the network</u>

>**Bra001852**.1-P=AT3G22120 CWLP | CWLP (CELL WALL-PLASMA MEMBRANE LINKER PROTEIN); lipid binding

>**Bra003006**.1-P=Uncharacterized protein; AT5G54170 INVOLVED IN: response to wounding

>**Bra001958**.1-P=Uncharacterized protein; AT2G16060 - GLB1, AHB1, ARATH GLB1, NSHB1, ATGLB1 | AHB1 (ARABIDOPSIS HEMOGLOBIN 1); oxygen binding/ oxygen transporter; Belongs to the globin family

>**Bra008269**.1-P=Uncharacterized protein; AT1G76640 | calmodulin-related protein, putative

>Bra012684.1-P= Uncharacterized protein; AT4G17030, ATEXLB1, (ARABIDOPSIS THALIANA EXPANSIN-LIKE B1

>**Bra012764**.1-P=Uncharacterized protein; AT4G15800, RALFL33 | RALFL33 (ralf-like 33); signal transducer

>**Bra019369**.1-P=Uncharacterized protein; AT4G22620 | auxin-responsive family protein

>**Bra019711**.1-P=Uncharacterized protein; AT1G66700, PXMT1 | PXMT1; S-adenosylmethionine-dependent methyltransferase

>**Bra029933**.1-P=Peroxidase; AT3G49120, ATPERX34, PERX34, PRXCB, ATPCB | PRXCB (PEROXIDASE CB); peroxidase; Belongs to the peroxidase family. Classical plant (class III) peroxidase subfamily

>**Bra031940**.1-P=Uncharacterized protein; AT5G64000, SAL2, ATSAL2 | SAL2; 3'(2'),5'-bisphosphate nucleotidase/ inositol or phosphatidylinositol phosphatase

>**Bra036984**.1-P=Uncharacterized protein; AT4G33710 | pathogenesis-related protein, putative

| **Primer Name** | **Sequence (5'->3')** | **Annealing temperature** | **Length (nt)** |
|---|---|---|---|
| Bra001852-FP | TGGTAAAACGCACGCGAAAG | 56 °C / 30 s | 20 |
| Bra001852-RP | TTTGAAGCCAGGTGGTGGAG | | 20 |
| Bra001958-FP | TGTTGTGAGTCAGCAGCACA | 56 °C / 30 s | 20 |
| Bra001958-RP | CTGGCACCGCCTCCTTTATT | | 20 |
| Bra003006-FP | TACGCGCTTACCAAGAGCAA | 60 °C / 30 s | 20 |
| Bra003006-RP | TCCGTTGTGTGTAACGGGAG | | 20 |
| GATA23-FP | GTGGTTCCTTGGAACACCCT | 56 °C / 30 s | 20 |
| GATA23-RP | TCAAAAGAACCAACCTTTGTGGG | | 23 |
| MAKR4-FP | GAGTGCAGCCTCCTTTTGGA | 60 °C / 30 s | 20 |
| MAKR4-RP | CGCAGGCTGTCTCCTCATAG | | 20 |

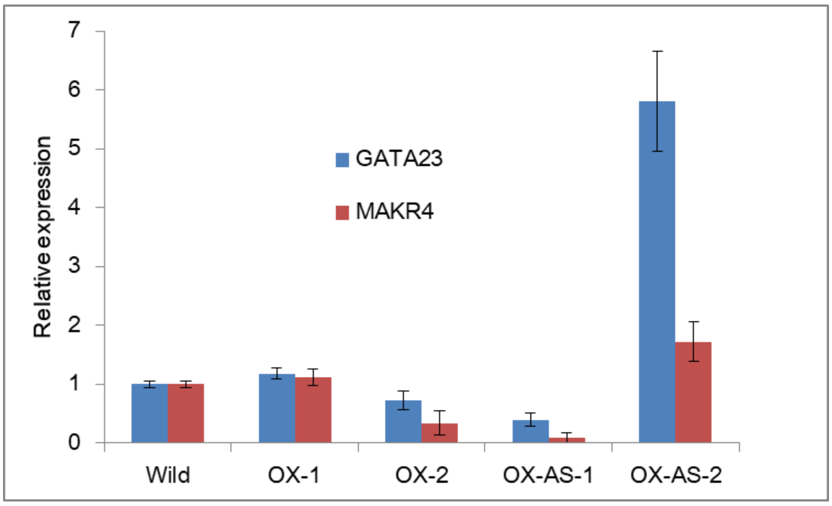


**Figure S4**. qRT-PCR based relative quantification of lateral root marker genes, *GATA23* and *MAKR4*.

**Table S1**. List of OJIP transient parameters collected from OX, OX-AS lines, and wild type *B. rapa* lines subjected to 8 days of progressive drought stress.

| BrEXLB1-AS lines | Replicate 1 | Replicate 2 | Replicate 3 | Average | SE |
|---|---|---|---|---|---|
| Bckg | 260 | 162 | 162 | 194.6666667 | 32.66666667 |
| Fo | 10631 | 12809 | 9851 | 11097 | 885.119201 |
| Fj | 27211 | 29553 | 27082 | 27948.66667 | 803.0305792 |
| Fi | 45613 | 51660 | 45515 | 47596 | 2032.196923 |
| Fm | 58194 | 64242 | 56634 | 59690 | 2320.124135 |
| Fv | 47563 | 51433 | 46783 | 48593 | 1437.741284 |
| Vj | 0.349 | 0.326 | 0.368 | 0.347666667 | 0.01214267 |
| Vi | 0.735 | 0.755 | 0.762 | 0.750666667 | 0.008089774 |
| Fm/Fo | 5.474 | 5.015 | 5.749 | 5.412666667 | 0.214095254 |
| Fv/Fo | 4.474 | 4.015 | 4.749 | 4.412666667 | 0.214095254 |
| Fv/Fm | 0.817 | 0.801 | 0.826 | 0.814666667 | 0.007310571 |
| Mo | 0.67 | 0.632 | 0.673 | 0.658333333 | 0.013195117 |
| Area | 15395077 | 11822609 | 16132228 | 14449971.33 | 1330804.584 |
| Fix Area | 57153324 | 62947368 | 55591720 | 58564137.33 | 2237497.491 |
| Sm | 323.678 | 229.864 | 344.831 | 299.4576667 | 35.32855871 |
| Ss | 0.52 | 0.515 | 0.548 | 0.527666667 | 0.010268615 |
| N | 621.976 | 446.33 | 629.825 | 566.0436667 | 59.89970281 |
| Phi_Po | 0.817 | 0.801 | 0.826 | 0.814666667 | 0.007310571 |
| Psi_o | 0.651 | 0.674 | 0.632 | 0.652333333 | 0.01214267 |
| Phi_Eo | 0.532 | 0.54 | 0.522 | 0.531333333 | 0.005206833 |
| Phi_Do | 0.183 | 0.199 | 0.174 | 0.185333333 | 0.007310571 |
| Phi_Pav | 939.374 | 912.764 | 944.399 | 932.179 | 9.815282726 |
| Pi_Abs | 3.556 | 3.43 | 3.684 | 3.556666667 | 0.073324242 |
| ABS/RC | 2.351 | 2.425 | 2.211 | 2.329 | 0.062748174 |
| TRo/RC | 1.922 | 1.942 | 1.826 | 1.896666667 | 0.035801924 |
| ETo/RC | 1.252 | 1.31 | 1.154 | 1.238666667 | 0.045524108 |
| DIo/RC | 0.43 | 0.484 | 0.385 | 0.433 | 0.028618176 |

| BrEXLB1-Sense lines | Replicate 1 | Replicate 2 | Replicate 3 | Average | SE |
|---|---|---|---|---|---|
| Bckg | 162 | 162 | 162 | 162 | 0 |
| Fo | 10143 | 10404 | 8811 | 9786 | 493.2879484 |
| Fj | 27667 | 27960 | 24481 | 26702.66667 | 1114.048822 |
| Fi | 45320 | 47108 | 42037 | 44821.66667 | 1484.925625 |
| Fm | 55301 | 58617 | 55626 | 56514.66667 | 1055.345178 |
| Fv | 45158 | 48213 | 46815 | 46728.66667 | 882.9583481 |
| Vj | 0.388 | 0.364 | 0.335 | 0.362333333 | 0.01532246 |
| Vi | 0.779 | 0.761 | 0.71 | 0.75 | 0.020663978 |
| Fm/Fo | 5.452 | 5.634 | 6.313 | 5.799666667 | 0.261988761 |
| Fv/Fo | 4.452 | 4.634 | 5.313 | 4.799666667 | 0.261988761 |
| Fv/Fm | 0.817 | 0.823 | 0.842 | 0.827333333 | 0.007535103 |
| Mo | 0.757 | 0.663 | 0.556 | 0.658666667 | 0.058064141 |
| Area | 15227274 | 16646788 | 16199915 | 16024659 | 419042.9565 |
| Fix Area | 54330196 | 57583580 | 54482664 | 55465480 | 1059964.202 |
| Sm | 337.2 | 345.276 | 346.041 | 342.839 | 2.828135251 |
| Ss | 0.512 | 0.549 | 0.603 | 0.554666667 | 0.026421792 |
| N | 658.159 | 629.112 | 574.336 | 620.5356667 | 24.57464108 |
| Phi_Po | 0.817 | 0.823 | 0.842 | 0.827333333 | 0.007535103 |
| Psi_o | 0.612 | 0.636 | 0.665 | 0.637666667 | 0.01532246 |
| Phi_Eo | 0.5 | 0.523 | 0.56 | 0.527666667 | 0.017476969 |
| Phi_Do | 0.183 | 0.177 | 0.158 | 0.172666667 | 0.007535103 |
| Phi_Pav | 944.214 | 941.554 | 934.562 | 940.11 | 2.878317101 |
| Pi_Abs | 2.937 | 3.653 | 5.355 | 3.981666667 | 0.717100024 |
| ABS/RC | 2.39 | 2.215 | 1.972 | 2.192333333 | 0.121197268 |
| TRo/RC | 1.952 | 1.822 | 1.66 | 1.811333333 | 0.084461694 |
| ETo/RC | 1.194 | 1.159 | 1.104 | 1.152333333 | 0.026193723 |
| DIo/RC | 0.438 | 0.393 | 0.312 | 0.381 | 0.036864617 |

| Wild type lines | Replicate 1 | Replicate 2 | Replicate 3 | Average | SE |
|---|---|---|---|---|---|
| Bckg | 292 | 162 | 162 | 205.33333 | 43.333333 |
| Fo | 11704 | 14565 | 10566 | 12278.333 | 1189.593 |
| Fj | 28285 | 38005 | 29032 | 31774 | 3122.9539 |
| Fi | 45743 | 61413 | 47174 | 51443.333 | 5001.9206 |
| Fm | 57675 | 72305 | 59853 | 63277.667 | 4557.2463 |
| Fv | 45971 | 57740 | 49287 | 50999.333 | 3503.6364 |
| Vj | 0.361 | 0.406 | 0.375 | 0.3806667 | 0.0132958 |
| Vi | 0.74 | 0.811 | 0.743 | 0.7646667 | 0.0231828 |
| Fm/Fo | 4.928 | 4.964 | 5.665 | 5.1856667 | 0.2398919 |
| Fv/Fo | 3.928 | 3.964 | 4.665 | 4.1856667 | 0.2398919 |
| Fv/Fm | 0.797 | 0.799 | 0.823 | 0.8063333 | 0.0083533 |
| Mo | 0.721 | 0.908 | 0.715 | 0.7813333 | 0.063357 |
| Area | 10798650 | 15030871 | 12274413 | 12701311 | 1240242.6 |
| Fix Area | 56748832 | 71139952 | 58776652 | 62221812 | 4497329.9 |
| Sm | 234.901 | 260.32 | 249.04 | 248.087 | 7.3532883 |

| | | | | | |
|---|---|---|---|---|---|
| Ss | 0.5 | 0.447 | 0.524 | 0.4903333 | 0.0227474 |
| N | 469.774 | 582.033 | 475.314 | 509.04033 | 36.531356 |
| Phi_Po | 0.797 | 0.799 | 0.823 | 0.8063333 | 0.0083533 |
| Psi_o | 0.639 | 0.594 | 0.625 | 0.6193333 | 0.0132958 |
| Phi_Eo | 0.51 | 0.474 | 0.515 | 0.4996667 | 0.0129142 |
| Phi_Do | 0.203 | 0.201 | 0.177 | 0.1936667 | 0.0083533 |
| Phi_Pav | 928.637 | 938.004 | 925.345 | 930.662 | 3.7920109 |
| Pi_Abs | 2.775 | 2.072 | 3.359 | 2.7353333 | 0.3720539 |
| ABS/RC | 2.509 | 2.8 | 2.318 | 2.5423333 | 0.140136 |
| TRo/RC | 2 | 2.236 | 1.909 | 2.0483333 | 0.0974412 |
| ETo/RC | 1.279 | 1.328 | 1.194 | 1.267 | 0.039145 |
| DIo/RC | 0.509 | 0.564 | 0.409 | 0.494 | 0.0453689 |
